# Impact of County-level health infrastructure on participation in a reform effort to reduce the use of jail for individuals with mental health disorders

**DOI:** 10.1186/s40352-023-00226-9

**Published:** 2023-07-04

**Authors:** Niloofar Ramezani, Maji Hailemariam, Alex J. Breno, Benjamin J. Mackey, Alison Evans Cuellar, Jennifer E. Johnson, Faye S. Taxman

**Affiliations:** 1 Department of Biostatistics, Virginia Commonwealth University, 830 East Main Street, PO Box 980032, Richmond, VA 23298-0032 USA; 2 Charles Stewart Mott Department of Public Health, College of Human Medicine, Michigan State University, Flint, MI USA; 3 Center for Advancing Correctional Excellence, Schar School of Policy and Government, George Mason University, Fairfax, VA USA; 4 Department of Health Administration and Policy, George Mason University, Fairfax, VA USA

**Keywords:** Underserved populations, Health disparities, Mental health, Care delivery, Mental health in jail, Criminal legal

## Abstract

**Background:**

The national Stepping Up Initiative has attracted over 500 counties interested in reducing the use of jail for individuals with mental health disorders. This paper identifies socioeconomic, criminal legal, and health care factors that predict the likelihood of counties joining Stepping Up.

**Results:**

After performing variable selection, logistic regression models were performed on 3,141 U.S. counties. Counties designated as medically underserved and/or mental health staffing shortage areas were less likely to participate in this initiative. Logistic regression models showed that larger counties (populations over 250,000) with better health care infrastructure, more mental health providers per capita, higher percent of Medicaid funded drug treatment services, and at least one medical school, were more likely to join Stepping Up. These counties had lower per capita jail populations, higher concentration of police resources, and higher pretrial incarceration rate.

**Conclusions:**

County-level health care delivery factors are major contributors to a county’s likelihood, or willingness, of engaging in Stepping Up reform efforts to reduce jail population with mental health disorders issues. Therefore, improving availability and accessibility of medical and behavioral health care in different communities, may facilitate efforts to address the unnecessary incarceration of individuals with mental health disorders.

**Supplementary Information:**

The online version contains supplementary material available at 10.1186/s40352-023-00226-9.

## Background

Mass incarceration is a public health crisis in the United States (Weidner and Schultz 2021). The U.S. has the world’s highest incarceration rate (Initiative pp 2018). People of color are disproportionately represented in justice settings of jails and prisons, probation and parole, and other forms of community supervision (Wildeman and Wang 2017; Blankenship et al. 2018). Every year, about two million people with serious mental illness (SMI) are booked into US jails (Wrenn et al. 2018). Different counties are allowed to use their own definition of SMI, but are encouraged to use the definition by National Institute of Mental Health (NIMH). Most jurisdictions base this definition on their state definition for SMI, which lines up with definitions for federal funding streams. According to the NIMH, some common characteristics of SMI include mental, behavioral, or emotional conditions that result in serious functional impairment. People with mental health disorders are more likely to be arrested, spend more time in pretrial detention, serve longer sentences, have lower chances of gaining parole, and have higher risk for mortality in jails (Wrenn et al. 2018). People with mental health disorders are disproportionately represented in US jails both in terms of raw numbers and duration of stay (Carroll 2016).

Reducing the number of people with serious mental health disorders in the criminal legal system has emerged as a major public health and safety priority. However, limited community-based programs and services can leave the criminal legal system as the main option for handling individuals with mental health disorders in many counties. It is estimated that less than 10% of justice-involved clients can access appropriate treatment services while in jail or in the community (Taxman et al. 2007). Strategies for decarcerating individuals with mental health disorders include police diversion to treatment in lieu of arrests (Lattimore et al. 2020), crisis intervention centers instead of jail (Hoffberg et al. 2020), specialized courts for individuals with mental health disorders and substance use disorders (Lowder et al. 2018), specialized reentry initiatives, and specialized probation mental health caseloads (Skeem et al. 2017; Skeem et al. 2018). But, limited use of standardized screening and assessment tools in jails and probation/parole settings still provides a challenge to diverting and treating people with mental health disorders (Lattimore et al. 2020). While Medicaid expansion has helped counties to expand access to existing substance abuse and mental health services (Kennedy-Hendricks et al. 2016), the lack of services still remains a challenge.

### Community mental health service capacity

The Health Resources and Services Administration (HRSA) designated shortage areas include Medically Underserved Areas (MUAs) as determined by the few primary care providers, higher rates of infant mortality, poverty, and/or large elderly population and the Health Professional Shortage Areas (HPSAs) specifically designates areas where there is a shortage of mental health providers. Of the 3,141 U.S. counties, 1025 (33%) of the counties are a mental health professional shortage area and 1461(45%) of the counties are designated as medically underserved. Only 535 of the U.S. counties are both medically underserved and mental health professional shortage areas. A disproportionate share of people with an untreated mental health disorder live in medically underserved and mental health (MH) personnel shortage counties (Bruckner et al. 2019). Because of the prevalence of behavioral health needs in these designated areas, the legal system has become the default mental health system (Carroll 2016). One effort at the national level to expand community-based services to keep individuals with behavioral health issues out of jail is the Stepping Up Initiative.

### The stepping up initiative

The Stepping Up Initiative is a national effort aimed to reduce the number of people with mental health disorders in jails. More than 500 counties (17 percent of U.S. counties) from across the U.S. have joined the initiative (Haneberg and Watts 2016; SU Initiative 2021). Varying number of counties from Alabama, Arizona, Arkansas, California, Colorado, Florida, Georgia, Hawaii, Idaho, Illinois, Indiana, Iowa, Kansas, Kentucky, Louisiana, Maine, Maryland, Massachusetts, Michigan, Minnesota, Mississippi, Missouri, Montana, Nebraska, Nevada, New Hampshire, New Mexico, New York, North Carolina, North Dakota, Ohio, Oklahoma, Oregon, Pennsylvania, South Carolina, South Dakota, Tennessee, Texas, Utah, Virginia, Washington, West Virginia, and Wisconsin currently participate in this initiative. A map showing the distribution of the Stepping Up counties across the U.S. can be found on the Stepping Up website (https://stepuptogether.org/the-counties/#/). Stepping Up is led by the National Association of Counties (NACo), the Council of State Governments Justice Center (CSG), and the American Psychiatric Association Foundation (APAF). Counties join Stepping Up by passing a resolution to pursue reductions in the jail population through six steps: (1) convene a diverse team of community behavioral health and criminal legal administrators and policy-makers, (2) identify a mental health screening tool, (3) monitor the flow of individuals with mental health disorders through the legal system, (4) examine community and jail treatment service capacity, (5) develop measurable outcomes, and (6) track progress by collecting data on each part of the plan. The sponsoring agencies have developed over 16 web-based tool-kits, offer ongoing webinars, and provide ongoing guidance to counties and states on implementation issues to reduce the use of the jail for individuals with mental health disorders. NACo-CSG-APAF host a technical assistance platform to assist counties (and states) in redesigning their legal and behavioral health systems, a platform that is available to both Stepping Up and non-Stepping Up counties. The six-step process with complementary policy design and implementation materials are offered at no cost to the counties, which makes reform strategies easily accessible. The reform framework for Stepping Up uses a change team approach which specifies that a team of actors from diverse agencies can achieve consensus on how best to achieve new goals such as reducing the number of individuals with mental health disorders in jail. The change team model has been experimentally tested and found to have impacts on increasing access to substance use services, improving access to treatment for youth in the juvenile justice services, and changing attitudes toward providing HIV services, to name a few (McCarty et al. 2007; Belenko et al. 2022; Visher et al. 2014) The unique aspect of this change team approach is that the six step procedures were designed by the aforementioned national association agencies and then each county can identify and implement the steps they desire. This differs from the above experimental randomized controlled trials in that this is an effectiveness study to understand how the change team approach works in this naturalistic, real world setting with little input from researchers. Stepping Up is a system reform effort and is not focused on adding a program. An evaluation of how well Stepping Up counties follow these steps and the impact on jail population and services in the community is currently being studied (Johnson et al. 2021). There are no existing studies on the impact of Stepping Up but the value of this study is that there is very little information in the literature on the characteristics of counties that are willing to engage in reform efforts for their local jail for individuals with mental health disorders.

The question is what distinguishes counties that join efforts to reduce the incarceration of individuals with serious mental health disorders—specifically Stepping Up—and how do the county-level characteristics affect the potential for policy reforms and technical assistance to achieve reform goals generally. An understanding of the characteristics of counties can be useful in tailoring technical assistance and other policy and programmatic efforts to be more applicable to the needs of counties who may find it most challenging to engage in policy reform efforts. The present study identifies county-level factors that predict the likelihood of a county joining Stepping Up to address unnecessary incarceration of individuals with mental health disorders. This study will contribute to a growing body of research on county-level characteristics that affect readiness for policy reform (Cuellar et al. 2021; Weidner and Schultz 2019), as well as efforts to assist jurisdictions to achieve such reforms.

## Methods

This cross-sectional observational study uses variable selection and logistic regression methods to identify county characteristics that predict the likelihood of U.S. counties participating in the Stepping Up Initiative. All 3,141 U.S. counties were included in analyses.

### Data

The most recent data (2014, 2015, 2016, and 2019) from five primary sources were used. First, we used the Vera Institute’s Incarceration Trends Database to obtain county-level incarceration statistics (Vera Institute of Justice 2018). The raw count of pretrial and jail populations for each county along with population size of each county were used to calculate the per capita jail and pretrial rates. Second, we used the County Health Rankings & Roadmaps (CHRR) data to obtain county-level demographic and health and health care delivery measures for each county (University of Wisconsin 2016) from the U.S. Census and American Community Survey (Census Bureau 2016). Third, we added police and crime data from the Uniform Crime Report (Census Bureau 2016; Federal Bureau of Investigation 2011). The most recent collected data on the number of police officers is from 2018. The numbers do not differ substantively per city and county from the 2011 data; however, there is a lot of missing data in the reported 2018 data. Therefore, for this variable, the 2011 data with much fewer missing data was used. Finally, from the Health Resources and Services Administration (HRSA; https://www.hrsa.gov), we obtained information about whether a county was a health professional shortage area for mental health and/or whether a county was designated medically underserved.

The above data sources were linked by county and state identifiers. The year each data set was collected is specified in Table 1. The study combines data collected from various years given availability of the most recent data. We used CHRR data since it was the most complete data file at the county level. The list of Stepping Up counties was extracted from The Stepping Up Initiative records (SU Initiative 2021) and a binary variable was created to indicate whether the county registered as a Stepping Up county or not.

**Table 1:** Model Variable Description

Variable	Source (year)
***County demographics***	
Size. Indicator variables were created for the three county populations: < 250,000, between 250,000 and 750,000, and over 750,000).	U.S. Census Population Estimates (2016)^a^
Income inequality which reflects the difference between the 80^th^ and 20^th^ income percentiles	American Community Survey, 5-year estimates (2016)^a^
High school graduation rate	EDFacts (2015), in the RWJ database
Percent of population that are Black	U.S. Census Population Estimates (2016)^a^
A designated mental health professional shortage area	Health Resources and Services Administration (HRSA; https://www.hrsa.gov)
Designated medically underserved county	Health Resources and Services Administration (HRSA; https://www.hrsa.gov)
***Health care related variables, population adjusted***	
Number of physically unhealthy days or days an individual indicates they were not feeling well	Behavioral Risk Factor Surveillance System (2016)^a^
Primary care physician rate based on number of physicians in a county	Area Health Resource File/American Medical Association (2015)^a^
Community MH centers per capita to indicate outpatient services	American Health Resources File (2019)^a^
Health care expenditure or total amount of costs from health care: Total Medicare expenditures per year	Dartmouth Atlas of Health Care (2015)^a^
Percent of drug treatment services paid by Medicaid^b^	IMS Institute for Healthcare Informatics (2016), in amfAR Opioid and Health Indicator database
Presence of one or more medical schools in the county	Association of American Medical Colleges (AAMC)
Mental health providers per capita (psychiatrists, psychologists, counselors, and social workers)	CMS, National Provider Identification (2017)^a^
***Crime-related variables***	
Number of police officers per total county population	Uniform Crime Report (2011)^c^
***Key Stepping Up indicators, population adjusted: ***Jail population per capita (average daily number of individuals in jail divided by the total county population)	Bureau of Justice Statistics (2015), in the VERA Incarceration trends report
***Key Stepping Up indicators, population adjusted: ***Jail pretrial population per capita (average daily number of individuals in jail awaiting trial divided by the total county population)	Bureau of Justice Statistics (2015), in the VERA Incarceration Trends Report

^a^_Reported in RWJ County Health Rankings and Roadmaps (https://www.countyhealthrankings.org/.)_

^b^_Percentage of buprenorphine and buprenorphine/naloxone combination prescriptions covered by Medicaid_

^c^_The most recent collected data on the number of police officers per county is from 2018. The numbers are not very different per city and county from the 2011 data but there is a lot more missing data in 2018. The team decided to use the older version of this data with much fewer missing data_

### Variables

The selection of variables for our models proceeded in stages. First, the study commenced with the research team identifying potential descriptors and then using best subset variable selection method (aka. all subset selection) to identify important non-collinear variables to predict Stepping Up participation of the U.S. counites. The number of mental health providers (psychologists, counselors, social workers, and psychiatrists) in a county and the size of the jail population adjusted for county’s population were the key variables related to the main goals of the Stepping Up initiative. The former gauges available mental health resources. The latter details the size of the incarcerated population. Pre-trial jail population is adjusted for county population and is considered part of defining jail capacity. The review of several demographic, economic, criminal legal, and health variables identified variables which were considered as potential predictors in this study. Details regarding these range of variables are described in prior work. (Johnson et al. 2021; Ramezani et al. 2022)

Next, instead of using the more than 50 predictor variables available in this study, we created a parsimonious model to predict the outcome of interest; it is well known that model parsimony benefits interpretability and helps avoid overfitting (Pedhazur 1982).^1^ For this purpose, best subset variable selection was used. This approach aims to find the subset of predictor variables achieving an optimal balance between model parsimony and model fit by evaluating all possible combinations of predictor variables (James et al. 2013). We also used machine learning techniques to double check the variable selection results and the resulted list of the selected variables agreed with the result of the best subset selection. The selected variables, and how they were standardized to be included in our models, are described in Table 1. Deviance and Akaike information criterion (AIC) were used to evaluate the fitting abilities of the models.

The selected variables included an indicator variable with three categories for the size of each county. The U.S. counties were categorized in three different sizes based on their population: Counties with a population less than 250,000 were categorized as small counties (*N=*2,880); counties with a population between 250,000 and 750,000 were categorized as medium-sized counties (*N=*186); and counties with a population greater than 750,000 were categorized as large counites (*N=*75). Data on small counties tend to contain more missing demographic and crime-related data elements. The size of the county is featured in all parts of the analyses. Some other variables are high school graduation rate (to measure the level of general education in a county which could be related to the outcome), income inequality ratio to measure socio-economic status of the county (which reflects the difference between the 80th and 20th income percentiles in a county), and percent of population that are African American in each county. The police officer rate per county population was used to capture the concentration of police as an indicator of the degree to which communities are policed. County- and city- level sworn-in police officers were added together and divided by the total population of the county.

### Statistical analysis

Statistical analyses were performed in R (Team R 2016). A significance level of $\alpha =.05$ was used and hypothesis tests were 2-sided. Preliminary bivariate correlations, summary statistics, and regression models identified demographic, health, health care delivery and criminal legal variables that predicted the outcome of interest. Models evaluated the observed differences between counties that adopted Stepping Up or not. For the main analysis, variable selection and logistic regression models were fitted to identify significant factors that differentiate Stepping Up from non- Stepping Up counties, which is the main outcome of this study (i.e., Stepping Up or non- Stepping Up). First, as explained in the Variables subsection, best subset variable selection, which looks at all possible combination of predictors, was applied to choose the best combination of predictors that predict the response variable. To ensure that the selected subset of independent variables was the optimum set to predict whether counties join Stepping Up or not, we double checked the variable selection results. This was done by performing two popular statistical machine learning approaches on all predictor candidates to detect and rank the order in which predictors interact, while simultaneously predicting the likelihood of being part of the Stepping Up Initiative. These two approaches are random forest and least absolute shrinkage and selection operator (LASSO) used to replicate the variable selection technique (Kukreja et al. 2006). Random forests and LASSO are dimension reduction and variable selection approaches appropriate for working with numerous predictor variables. These methods allowed us to select the most relevant variables among the predictors. LASSO is one of the most widely used methods to select relevant variables when there is a high number of variables—which describe the highest percentage of the response variation—for inclusion (Tibshirani 1996; Zhang et al. 2016). The list of the selected predictors for inclusion in the logistic regression model agreed with the selected predictors from best subset selection method. The variable selection step helped to prevent serious multicollinearity issues in advance of fitting the logistic regression models.^2^ To understand why some counties are willing to participate in the Stepping Up Initiative while other counties are not willing to commit to this effort, logistic regression model including the selected variables was performed to identify significant factors that best estimate the likelihood of counites participating in the Stepping Up Initiative.

## Results

**Fig. 1:**
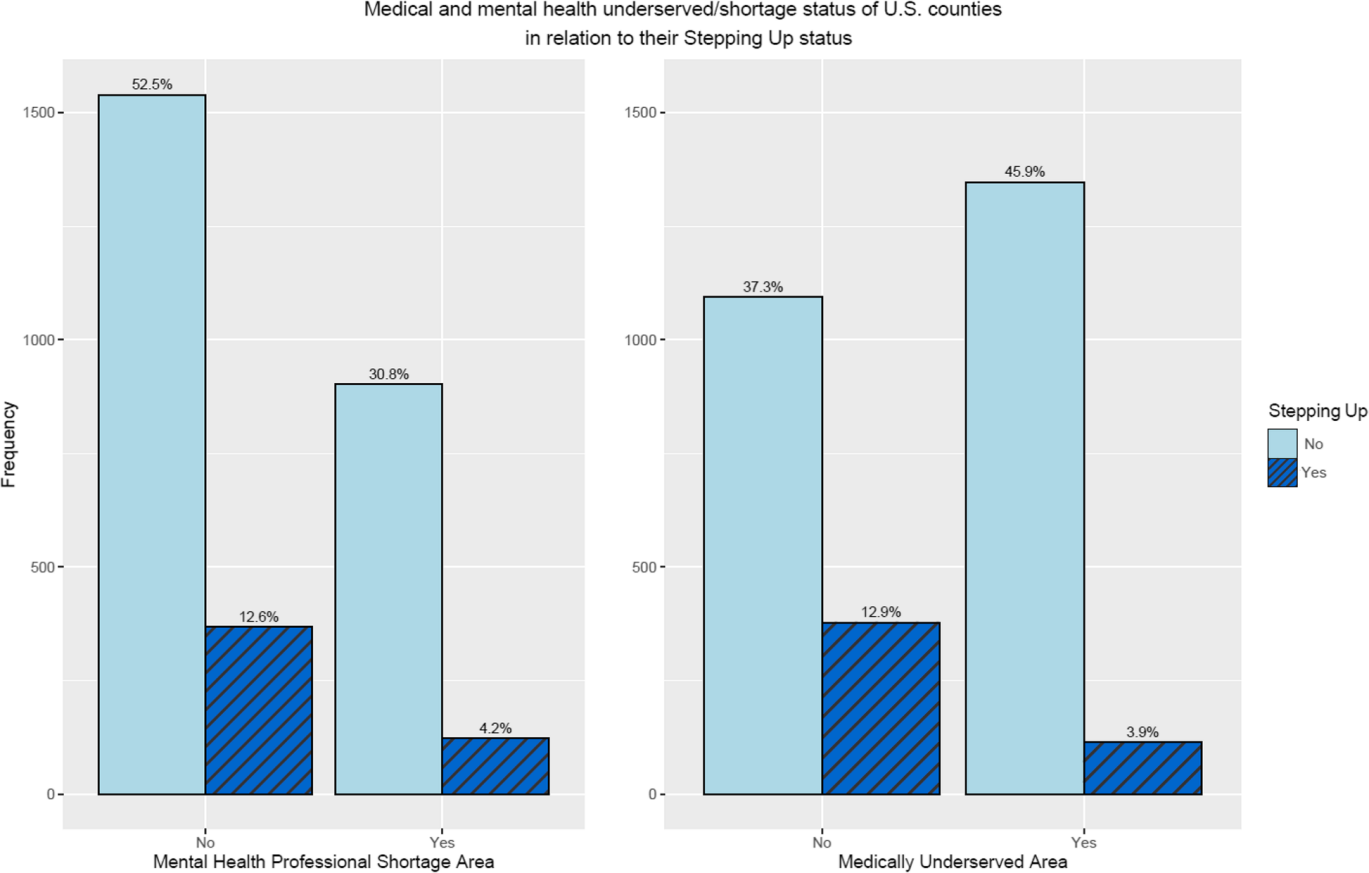
Bar plots of medical and mental health underserved/shortage status of U.S. counties in relation to their Stepping Up status

Correlation and logistic regression analyses confirmed that the counties that are classified as medically underserved and/or HRSA mental health shortage areas are less likely to participate in the Stepping Up initiative. Figure 1 shows this relationship and Figure 1S in the appendix illustrates the correlation analysis result. From the 1025 counties in a mental health professional shortage area, 123 (12%) joined Stepping Up initiative versus 21% of counties that were not within mental health professional shortage areas which joined the initiative. This would mean overall, only 4.2% of all the U.S. counties are in a designated mental health professional shortage area and have joined the Stepping Up. From the 1461 medically underserved counties, 114 (7.8%) have joined Stepping Up initiative versus 28% of counties that were not medically underserved which joined the initiative. This would mean overall, only 3.9% of all the U.S. counties are in a medically underserved area and have joined the Stepping Up initiative. These percentages of medically underserved and mental health professional shortage counties that join Stepping Up initiative are shown in Fig. 1. The frequency of counties in each category is shown on the Y-axis of this bar chart and their overall percent is written above each bar. Therefore, in the next phase of the analysis, variables related to medical and mental health providers/services of the counties, such as primary care provider rate, mental health provider rate, and community mental health centers per capita, needed to be considered while attempting to identify factors that could potentially predict the likelihood of the U.S. counties joining the Stepping Up initiative. This gave us the opportunity to control for the shortage (or availability) of medical and mental health services in the communities, while predicting counties joining the Stepping Up initiative or not.

### Logistic regression models

Table 1 illustrates the variables used to predict the likelihood of Stepping Up participation of the U.S counites. Accounting for the variables related to medically underserved and/or HRSA mental health shortage status of the counties, the effects of the following variables on counties participation in Stepping Up Initiative are tested within a logistic regression: 1) demographics such as county size, high school graduation rate, income inequality ratio, proportion of the population that is Black; 2) health care infrastructure such as average number of physically unhealthy days within a given month (30 days), primary care physicians per capita rate, total health care costs, total health care expenditures, proportion of drug treatment services paid by Medicaid, community MH centers per capita, presence of a medical school in the county, and mental health provider rate; and, 3) legal system factors including police officers per capita rate and per capita jail population. Predictor variables came from different scales and were scaled/standardized.

### Findings

Table 2 shows the results of the fitted logistic regression model on the aforementioned predictor variables while predicting the odds of counties being classified as Stepping Up. Estimated coefficients, odds ratios (OR), OR 95% confidence intervals, standard errors, and p-values for the test of significance of each variable are included in Table 2. The OR indicate how the odds of participating in Stepping Up effort changes as each predictor value varies, and the p-value column signifies whether each variable plays a statistically significant role in predicting the outcome. The direction of the relationship is designated by the value of the OR and whether the estimated coefficient is negative or positive.

**Table 2:** Logistic regression fitted to selected variables to predict Stepping Up participation of the U.S. counties

	Estimate	OR	95% CI of OR	Std. Error	*P*-value
(Intercept)	-2.14	.12	.10-.14	0.10	<0.001^a^
Mental health provider rate	.12	1.13	.98-1.31	0.07	0.0964
Jail population per capita	-1.68	.19	.09-.39	0.38	<0.001^a^
Jail pretrial population per capita	.56	1.75	1.09-2.83	0.24	0. 0217^b^
Average number of physically unhealthy days	-.34	.71	.60-.84	0.08	<0.001^a^
Primary care physician rate	.01	1.01	.88-1.17	0.07	0. 8547
High school graduation rate	-.18	0.83	.74-.94	0.06	0.0039^a^
Income inequality rate	.06	1.06	.91-1.25	0.08	0.4416
Health care costs	-.52	.59	.50-.70	0.08	<0.001^a^
Percent of Black Population	.01	1.01	.99-1.02	0.01	0.0596
Percent of drug treatment paid by Medicaid	.21	1.23	1.09-1.39	0.06	<0.001^a^
Police per capita	-.61	.54	.42-.71	0.13	<0.001^a^
Community MH centers per capita	-.01	.99	.83-1.18	0.09	0. 8963
Medical School Indicator	.88	2.41	1.39-4.19	0.28	0.0017^a^
County size Medium vs Small^c^	1.13	3.10	2.13-4.51	0.19	<0.001^a^
County size Large vs Small^c^	1.82	6.15	3.12-12.10	0.34	<0.001^a^

^a^_Represents significance at the 0.01 level in a two-tailed test_

^b^_Represents significance at the 0.05 level in a two-tailed test_

^c^_County size small is the comparison category for the county size variable and no medical school is the comparison category for the medical school indicator_

As shown in Table 2, county-level public health factors are key contributors influencing whether a county is involved in Stepping Up. Notably, lower average number of physically unhealthy days within a given month (OR=.71; *p<*.001), lower health care costs (OR=.59; *p<*.001), higher percent of drug treatment paid by Medicaid (OR=1.23; *p<*.001), and presence of a medical school in the county (Or= 2.4; p=.002) were among the important factors contributing to a higher likelihood of counties being classified as Stepping Up. Smaller jail population per capita (OR=.19; *p<*.001), higher per capita rate of pretrial jail population (OR=1.75p=.022), and lower per capita rate of police officers (OR=.54; *p<*.001) were important criminal legal system variables contributing to higher odds of counties being Stepping Up. Finally, among demographic variables, counties with a lower high school graduation rate (OR=.83; p=.004) and medium- or large-sized population (OR=3.10; *p<*.001, OR=6.15; *p<*.001, respectively) were more likely to be among the Stepping Up counties.

For the sake of sparsity, a logistic regression with fewer selected variables was performed, based on a more parsimonious LASSO fit. Table 3 shows the smaller fitted logistic regression model to estimate the odds of counties being Stepping Up or not in a more parsimonious model. This model resulted in a poorer model fit due to a smaller number of predictors (AIC=2157.7 and residual deviance=2137.7 compared to the previous model with AIC=2008.8 and residual deviance=1976.8).^3^ Once again, higher rate of mental health provider (OR=1.20; p=.002), lower average number of physically unhealthy days within a given month (OR=.83; p=.008), lower health care costs (OR=.63; *p<*.001), and higher percent of drug treatment services paid by Medicaid (OR=1.23; *p<*.001) were among important health-related factors contributing to a higher likelihood of counties joining Stepping Up. Lower rate of per capita jail population (OR=.45; *p<*.001) and lower rate of per capita police officers (OR=.61; *p<*.001) were among important criminal legal system variables that contributed to higher odds of counties being Stepping Up. Large- and medium-sized counties (OR=4.50; *p<*.001 and OR=14.06; *p<*.001, respectively) are more likely to participate in the Stepping Up.

**Table 3:** Logistic regression fitted--more parsimonious list of variables predicting Stepping Up participation

	Estimate	OR	95% CI of OR	Std. Error	Pr(>|z|)
(Intercept)	-2.05	0.13	.11-.15	0.5498	<0.001^a^
Mental health provider rate	0.18	1.20	1.07-1.35	39.5819	0.0021^a^
Jail population per capita	-0.79	0.45	.29-.71	24.2956	<0.001^a^
Average number of physically unhealthy days	-0.18	0.83	.72-.95	0.0973	0.0079^a^
Primary care physician rate	0.06	1.06	.95- 1.20	0.0017	0.2926
Health care costs	-0.47	0.63	.54-.72	0.0001	<0.001^a^
Percent of drug treatment paid by Medicaid	0.21	1.23	1.10-1.38	0.0043	0.0003^a^
Police per capita	-0.50	0.61	.48-.76	0.0845	<0.001^a^
County size Medium vs Small^b^	1.50	4.50	3.16-6.41	0.1807	<0.001^a^
County size Large vs Small^b^	2.64	14.06	7.78-25.41	0.3018	<0.001^a^

^a^_Represents significance at the 0.01 level in a two-tailed test_

^b^_County size small is the comparison category for the county size variable and no medical school is the comparison category for the medical school indicator_

## Discussion

This study evaluated the distinguishing characteristics of counties that join a mental health decarceration policy reform effort (Stepping Up) from counties that do not. Approximately 28 percent of the 3,141 counties in the U.S. are involved in some kind of initiative to improve mental health and/or substance use care for justice-involved individuals (such as Stepping Up, McArthur Safety and Justice Challenge or other programs). Around 17% of the U.S. counties are focused in Stepping Up effort that focuses on justice-involved individuals with behavioral health issues (Cuellar et al. 2021). This study helps us understand what county characteristics predict an interest and willingness to examine non-incarcerative options for individuals with mental health disorders in one such effort. Counties in mental health professional shortage areas and/or medically underserved areas are less likely to participate in Stepping Up, as expected. In addition, specific county-level health care system factors predicted counties’ likelihood of engaging in Stepping Up efforts. Counties with healthier populations and better public health infrastructures appear to be more likely than other counties to join reform efforts to reduce the number of people with serious mental health disorders in jail. Specifically, counties with better health indicators and greater health care access (as determined by higher mental health provider rate, higher primary care physician rate, lower health care costs, higher percent of drug treatment paid by Medicaid, and fewer physically unhealthy days) and less reliance on the legal system (smaller jail population and fewer police per capita) are more likely to join Stepping Up. Counties with medium- or large-sized populations were more likely to join Stepping Up than those with smaller (less than 250,000) populations.

Overall, a stronger infrastructure with access to a broader array of medical, behavior health, and justice resources may help counties to engage in decarceration policy reform efforts like Stepping Up. Jurisdictions with health service deserts were more likely to occur in non-Stepping Up counties. These counties may find it more challenging to expand community-based behavioral health alternatives to reduce the use of jail for individuals with mental health disorders. A related challenge in less populous counties and/or counties with fewer health services is the limited legal and/or behavioral health personnel to work on reform activities and to meet community behavioral health needs. In these counties, workforce resources to support strategic policy efforts may be needed. Many counties do not have dedicated mental health facilities or programming/planning staff to help build service capacity in the community. In this study, counties which are ready to engage in legal policy reform—as evidenced by the commitment of political leadership in a county through a Stepping Up resolution—are more likely to be counties with greater public health-related resources, lower jail populations, and smaller police per capita.

In terms of criminal legal characteristics, violent crime rate in a county was unrelated to enrollment in Stepping Up. Counties with more police per capita were less likely to join Stepping Up. More police and safety resources in a community may reflect lower political will to reform the use of jail. Overall, though, health system factors were stronger predictors of counties joining Stepping Up than criminal legal factors. Overuse of the justice system for individuals with mental health disorders and inability to participate in efforts to address this problem reflect community public health deficits. While efforts to re-examine the role of police and arrest practices are being discussed to address overuse of the justice system (John Jay College Research Advisory group on Preventing and Reducing Community Violence 2020), it appears that concentration of police has broader impacts on legal system reform such as decarceration.

Although race was not statistically significant in the logistic regression models in the presence of other factors, counties with a higher percent of Black population trended toward being more likely to join Stepping Up. This is important because communities of color experience a disproportionate double-burden of incarceration and mental health disorders due to the impact of poor health infrastructure and systemic racism. (Schnittker et al. 2011; Sugie and Turney 2017)

The county-level characteristics differentiating Stepping Up and non-Stepping Up counties may suggest the need for different pathways or supports for reforms depending on county characteristics, particularly given the importance of community mental health service capacity. Stepping Up’s approach to advancing policy reforms at the county level is to ask counties to form a multi-agency change team, to create infrastructures to track metrics related to decarceration and service delivery, and then to consider ways to keep people out of jail (e.g., enhancing screening and assessment before incarceration, providing therapeutic walk-in centers, or providing transitional housing supports) (National Association of Counties 2021). Some counties may be better equipped to follow this recipe than others. For example, a data-driven strategy like Stepping Up requires a strong data analytical infrastructure—one that also links management information systems across the justice and health spectrum. Stepping Up measures require the ability to measure the number of individuals with behavioral health needs moving in and out of jail and those that are connected to community treatment. Such data metrics are a challenge for many counties because the information does not exist in a management information system, the data systems cannot be linked, and/or the proper consents or releases are not typically used. Preliminary steps to help counties that have yet to begin to unravel their mass incarceration policies could be to focus on providing data resources to augment existing management information systems, to implement screening tools across agencies that can identify individuals with mental health disorders, and to build data analysis capacity. Or perhaps building mental health treatment capacities might facilitate greater progress. These ingredients are needed to document the need for policy changes as well as measure progress in policy reform efforts.

The study’s cross-sectional design is a potential limitation. The design limits the ability to know whether better pre-existing health infrastructure assisted counties in joining Stepping Up, whether Stepping Up helped counties address service deserts and improve access to and availability of community-based services, neither, or both. A second potential limitation is the use of county-level measures instead of micro-geographical measures to explain the availability of health and behavioral health (i.e., mental health, substance use, etc.) services. Future work will address these limitations.

## Conclusions

Most counties that have not yet joined Stepping Up are rural, have smaller populations, are in mental health professional shortage areas, are designated as medically underserved, and/or have limited community health infrastructure. Efforts to build more robust behavioral health care delivery systems and improving community capacity for mental health and related services may help increase participation in legal system reform efforts in less populous counties. Counties that lack health and infrastructure resources may need additional supports and/or a different prescription for policy teamwork such as banding counties together, engaging citizen groups in policy teams to advance efforts to expand services, working on smaller goals, and obtaining state-level support. This type of county-level technical assistance is more directive and can probably facilitate change for smaller counties or those with fewer resources. The characteristics of the counties should influence the type of technical assistance needed to facilitate decarceration efforts. More studies are needed to explore the types of assistance that are useful for counties to make changes in how they use the legal system for individuals with mental health disorders.

Jail incarceration may detrimentally impact the physical and behavioral health of a community (Kajeepeta et al. 2021; Kajeepeta et al. 2020; Nosrati et al. 2019). The lack of community mental health services, and/or substance use services, has been attributed to deinstitutionalization of mental health services in the 1980s (Fisher et al. 2006). Limited community mental health services restrict the options communities have to respond to individuals with mental health disorders who commit crimes—even minor offenses like disorderly conduct (Fisher et al. 2006). Therefore, initiatives such as Stepping Up, which aim at reducing the use of jail for individuals with mental health disorders, could become important to communities. This means identifying the factors that predict the likelihood of counties joining an initiative that addresses unnecessary incarceration of individuals with mental health disorders becomes crucial. In this study, we saw that larger counties with better health care infrastructure, more mental health providers per capita, higher percent of Medicaid funded drug treatment services, and at least one medical school, were more likely to join Stepping Up. Therefore, improving availability and accessibility of medical and behavioral health care of communities may facilitate efforts to help individuals with mental health disorders in jails.

## Supplementary Information

**Additional file 1: Figure 1S.** Mosaic plots of medical and mental health underserved/shortage status of U.S. counties in relation to their Stepping Up status.

## Data Availability

The datasets used and/or analyzed during the current study can become available from the corresponding author on reasonable request.

## Footnotes

1 Overfitting occurs when a model is too complex and begins to describe the random error in the data rather than the relationships between variables.

2 Multicollinearity occurs in the presence of high intercorrelations among two or more predictors.(Pedhazur 1982)

3 A smaller AIC and a larger residual deviance indicate a better fitting model.
