# Supplementary material for: Impact of County-level health infrastructure on participation in a reform effort to reduce the use of jail for individuals with mental health disorders
Source: Health Justice. 2023 Jul 4;11:27. doi: 10.1186/s40352-023-00226-9 (PMC10318809; doi:10.1186/s40352-023-00226-9)

**APPENDIX**

Figure 1S plots the number of units in each cell/category (Stepping Up or not versus medically underserved or not on the left plot; Stepping Up or not versus mental health professional shortage area or not on the right plot) and provides a measure of statistical significance for the relationship between categories using the Pearson residuals and the standard deviations. In this plot, a residual greater than 2 or less than -2 (the areas that are not in grey) represents a departure from independence significant at the 95% level. Different patterns and shades in each cell represent the level of residual in that cell/combination of levels. Diagonal stripes and dashed areas mean that there are more observations in their cells than would have been be expected under the independence hypothesis and horizontal stripes and black areas mean there are fewer observations than would have been expected in those cells. Collectively, the two plots show that counties that are medically underserved, as well as counties that are designated mental health professional shortage areas, are significantly less likely to participate in Stepping Up initiative (both overall p-values being less than .001). Variables used in this plot are provided in Table 1.

***Figure 1S.*** Mosaic plots of medical and mental health underserved/shortage status of U.S. counties in relation to their Stepping Up status


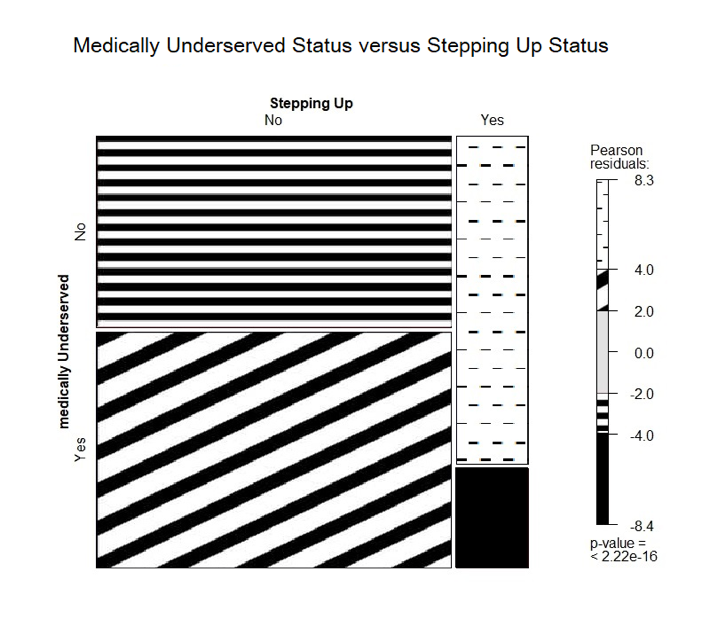

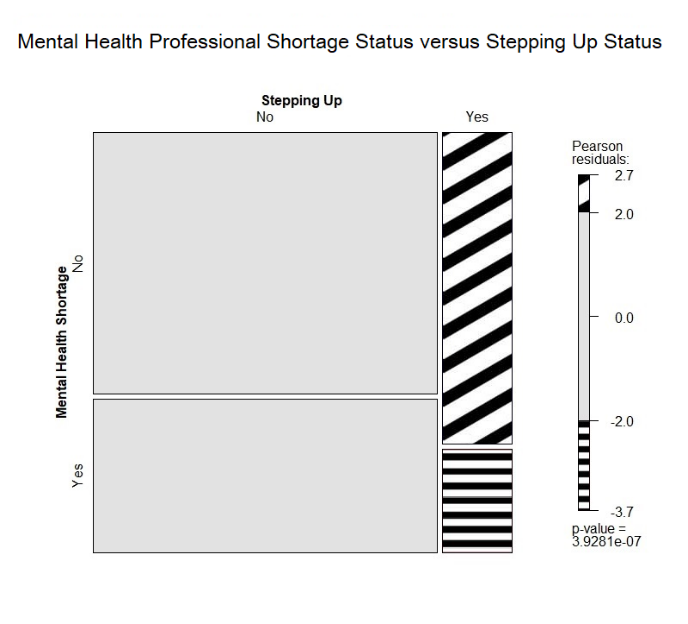

Supplement: Supplementary file 1 — Additional file 1: Figure 1S. Mosaic plots of medical and mental health underserved/shortage status of U.S. counties in relation to their Stepping Up status. [file 40352_2023_226_MOESM1_ESM.docx]
